# Biomimetic electrospun PVDF/self-assembling peptide piezoelectric scaffolds for neural stem cell transplantation in neural tissue engineering†

**DOI:** 10.1039/d4ra02309a

**Published:** 2024-07-05

**Authors:** Mahdi Forouharshad, Andrea Raspa, Giuseppe Fortino, Maria Gessica Ciulla, Arman Farazdaghi, Vlad Stolojan, Luca Stendardo, Silvia Bracco, Fabrizio Gelain

**Affiliations:** a Center for Nanomedicine and Tissue Engineering (CNTE), ASST Grande Ospedale Metropolitano Niguarda 20162 Milan Italy f.gelain@css-mendel.it; b Department of Biotechnology and Bioscience, University of Milano – Bicocca via R. Cozzi 55 20125 Milano Italy; c Chemical and Biomolecular Engineering Department, Whiting School of Engineering, Johns Hopkins University MD USA; d Advanced Technology Institute, Electrical and Electronic Engineering, University of Surrey Guildford GU2 7XH UK; e Department of Materials Science, University of Milano – Bicocca via R. Cozzi 55 20125 Milano Italy; f Institute for Stem-Cell Biology, Regenerative Medicine and Innovative Therapies, IRCCS Casa Sollievo della Sofferenza 71013 San Giovanni Rotondo Italy

## Abstract

Piezoelectric materials can provide *in situ* electrical stimulation without external chemical or physical support, opening new frontiers for future bioelectric therapies. Polyvinylidene fluoride (PVDF) possesses piezoelectricity and biocompatibility, making it an electroactive biomaterial capable of enhancing bioactivity through instantaneous electrical stimulation, which indicates significant potential in tissue engineering. In this study, we developed electroactive and biomimetic scaffolds made of electrospun PVDF and self-assembling peptides (SAPs) to enhance stem cell transplantation for spinal cord injury regeneration. We investigated the morphology and crystalline polymorphs of the electrospun scaffolds. Morphological studies demonstrated the benefit of using mixed sodium dodecyl sulfate (SDS) and SAPs as additives to form thinner, uniform, and defect-free fibers. Regarding electroactive phases, β and γ phases—evidence of electroactivity—were predominant in aligned scaffolds and scaffolds modified with SDS and SAPs. *In vitro* studies showed that neural stem cells (NSCs) seeded on electrospun PVDF with additives exhibited desirable proliferation and differentiation compared to the gold standard. Furthermore, the orientation of the fibers influenced scaffold topography, resulting in a higher degree of cell orientation in fiber-aligned scaffolds compared to randomly oriented ones.

## Abstract 2

Piezoelectric materials can provide *in situ* electrical stimulation without external chemical or physical support, opening new frontiers for future bioelectric therapies.

## Graphical abstract

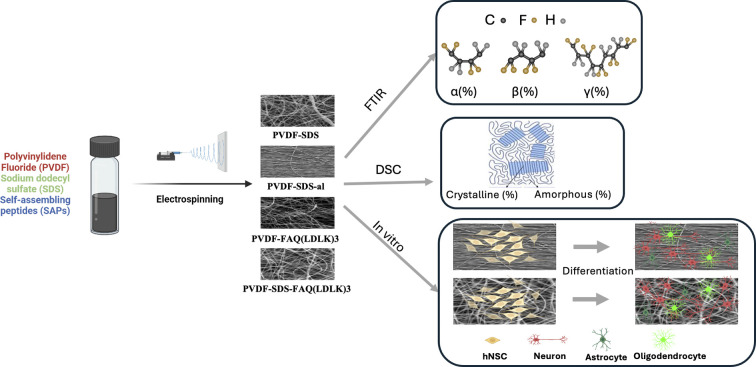

## 1. Introduction

Spinal cord injuries (SCIs), the leading cause of irreversible paralysis, have a profound and devastating impact on the lives of more than 20 million people worldwide due to the limited capability to regenerate or replace neuronal tissue.^1^ Previous studies in the field of neurobiology revealed that the deficiency of neuronal regeneration in the central nervous system (CNS) is not only because of the intrinsic lack of CNS axon regeneration ability but also because of extrinsic cell growth inhibition signals conferred by the CNS environment within the damaged tissue.^2^ Thus, if neurons are provided with the correct set of biological and physical stimuli, regeneration of the damaged neuronal tissue should be favored. Neural regeneration using Neural Stem Cells (NSCs) has demonstrated tremendous potential for neuro-regenerative therapies in humans by promoting angiogenesis and neurogenesis.^3^ Although some studies have shown promising cell survival, integration with host tissue, and synapse generation that all lead to improved recovery after SCIs, some limitations still exist, such as retention, poor engraftment, low neural plasticity, uncontrolled differentiation of transplanted stem cells, oxidative stress, lack of growth factors, and limited vascularization.^4–7^ Transplanted cells and regenerating nervous fibers require pro-regenerative substrates, favoring their engraftment and spatially guiding the host tissue regeneration, to regenerate the neural circuitry preexisting the injury. Advances in biomaterial design, manufacturing, and surface chemistry have vastly improved the safety and function range of implantable biomaterials such as cell scaffolding. Despite significant progress in neural regeneration on electrospun polymer scaffolds containing conventional neurotrophic factors such as nerve growth factor (NGF), the short half-life time and fast diffusion of factors like NGF significantly limit those scaffolds' efficiency *in vivo*.^8^ Electrical stimulation (ES) has been demonstrated to be a promising alternative to conventional growth factor treatments for the differentiation of various cell types, including neurons. This differentiation is mediated through multiple signaling pathways, including the MAPK/ERK pathway and the cAMP-dependent pathway.^9,10^ The nervous system (NS) is highly influenced by ES, which serves as the primary means of communication.^11^ Applying ES to scaffolds serves to mimic not only the electrical properties of the NS but also to reduce inflammatory response after implantation.^12^ Moreover, ES application improves the promotion of neural cells migration, proliferation, and differentiation, aiding the neurite extension process and resulting in a larger mean number of axons and an increased number of blood vessels in endoneurial and nervous areas.^13,14^

Piezoelectric materials, which can provide *in situ* ES without external chemical or physical support, open new frontiers for future bioelectric therapies. The native mechanisms of neuronal activation by ES are described by voltage-gated ion channels that ES causes both membrane potential depolarization and ion flow across the membrane *via* voltage-gated channels.^11^

Polyvinylidene fluoride (PVDF) serves as an electroactive biomaterial with the ability to enhance bioactivity through instantaneous ES. It can be tailored to create intelligent scaffolds that stimulate and regulate cell growth and behavior^15^ possesses piezoelectricity and processability, indicating significant potential in tissue engineering.^16^ This attribute is particularly advantageous in neural tissue engineering, enabling the development of scaffolds responsive to electrical cues, mimicking the native neural environment. Such electrical responsiveness plays a crucial role in influencing cell behavior and promoting neural regeneration.^17^

Electrospun fibrous PVDF scaffolds can effectively mimic the structure and components of the targeted extracellular matrix (ECM), both biochemically and electrically. This makes them promising scaffolds for neural tissue engineering. The high surface area and interconnected porous structure of these scaffolds create an environment conducive to cell adhesion, proliferation, and migration.^14^

The mechanical properties of electrospun PVDF scaffolds can be customized to match those of native neural tissue. This customization is essential for providing the necessary support and mechanical cues to cells during tissue regeneration. PVDF's flexibility and durability make it suitable for replicating the mechanical properties of neural tissue.^14^

Additionally, PVDF, as a hydrophobic polymer, can be modified or combined with other biomaterials to enhance its hydrophilicity, biochemical properties, and compatibility with neural tissue. Surface modifications, such as the incorporation of bioactive molecules or peptides, can promote specific cellular interactions and improve neural tissue regeneration. Preliminary *in vitro* studies on electrospun PVDF and PVDF-TrFE scaffolds have demonstrated the promotion of neural differentiation in PC12 cells compared to conventional *in vitro* differentiation protocols using NGF.^18^ Furthermore, hNSCs showed better differentiation into β-III tubulin (βIII-TUB)-positive cells and greater average neurite length,^19^ especially with low-aligned PVDF scaffolds.^20^ These scaffolds also facilitated the alignment and proliferation of Schwann cells and fibroblasts.^21,22^

A PVDF membrane was also used as an artificial nerve conduit for peripheral nerve injury repair. The PVDF scaffold revealed a sufficient scaffold biocompatibility with Schwann cells and no apparent cytotoxicity regarding neonatal rat and adult human Schwann cells along with enhanced bidirectional outgrowth of axons.^23^ On the other hand, fabricating a piezoelectric PVDF/graphene oxide scaffold *via* a non-solvent-induced phase separation method for nerve tissue engineering applications resulted in the improvement of attachment, spreading, and proliferation of PC12 cells.^24^

In this study, modified PVDF scaffolds with self-assembling peptides (SAPs) were manufactured to biomimic the microenvironment of the ECM for NSC growth and increase biodegradation by manipulating the scaffold surface's hydrophilicity which downregulates inflammatory response and promotes anti-inflammatory M2 macrophage growth.^25^ Biofunctionalized SAPs have been extensively investigated as NSCs transplantation carriers in neural tissue engineering and are appreciated for their excellent biocompatibility and biodegradability properties.^26,27^ On this basis, we explored the influence of solution parameters (co-solvent) and additives (SDS and SAPs) on the resulting PVDF nanofibers in terms of morphology, quantities of polymorph phases, and direct piezoelectric effect due to sound-induced strain rates at various sound frequencies. We investigated the role of the piezoelectric PVDF and SAPs as parameters beneficial to a pro-regenerative micro-environment by measuring the cell viability, differentiation, proliferation, and by the studying adhesion patterns of NSCs on the tested scaffolds.

## 2. Materials and methods

### 2.1 Materials and preparation

#### 2.1.1 Materials

Polyvinylidene fluoride (PVDF), with an average molecular weight of 275 000 Mw, dimethylformamide (DMF) (solvent), acetone (co-solvent), and sodium dodecyl sulfate (SDS) (anionic surfactant) were purchased from Merck (Merck Millipore, Darmstadt, Germany), Sigma Aldrich (Sigma Aldrich Chemie GmbH, München, Germany) and used as received. The linear sequence NH_2_-FAQRVPPGGG(LDLK)_3_-CONH_2_ peptide (dubbed FAQ(LDLK)_3_) was synthesized *via* microwave-assisted Fmoc SPPS on a 0.56 mmol g^−1^ Rink Amide 4-methylbenzhydrylamine resin (0.5 mmol g^−1^ substitution) using a CEM Liberty Blue system (CEM Corp., Matthews, NC, Canada) with a 0.25 mmol scale.^26^

#### 2.1.2 Preparation of the scaffolds using electrospinning

Electrospinning solutions for PVDF-based fibers were prepared by dissolving 25% w/v of PVDF in either pure solvent, DMF, or containing acetone with different ratios (100 : 0 and 60 : 40 v/v, DMF to acetone). The PVDF was first dissolved in the chosen primary solvent (DMF) *via* continuous stirring for 4 h at 70 °C. After cooling, acetone was added (if required), and the solution was stirred again for 2 h at room temperature. In the case of the solutions that included SDS, the concentrations of PVDF and SDS were 24.75% w/v and 0.25% w/v, respectively. In the case of the solutions that include only the peptide FAQ(LDLK)_3_, the concentrations of PVDF and FAQ(LDLK)_3_ were 23.75% w/v and 1.25% w/v, respectively. Finally, in the case of the solution which includes both SDS and FAQ(LDLK)_3_ peptide, the concentrations of PVDF, SDS, and FAQ(LDLK)_3_ were 23.5% w/v, 0.25% w/v, and 1.25% w/v, each. Table 1 summarizes the preparation conditions of the solutions and the electrospinning conditions used to produce the fibers.

**Table 1:** Optimal parameters for the manufacturing of the scaffolds: solution concentration (25% w/v), solvents, voltage (*V*), additives, flow rate (μL h^−1^), and distance (*D*)

Samples	PVDF (%)	FAQ(LDLK)_3_ (%)	SDS (%)	DMF (%)	Acetone (%)	Voltage (kV)	Distance (cm)	Flow rate (μL h^−1^)
PVDF	100	0	0	60	40	17	20	300
PVDF-SDS	99	0	1	60	40	17	20	300
PVDF-SDS-al	99	0	1	60	40	17	20	200
PVDF-DMF100	100	0	0	100	0	17	20	300/400
PVDF-SDS-DMF100	99	0	1	100	0	17	20	200/300
PVDF-FAQ(LDLK)_3_	95	5	0	60	40	17	20	300
PVDF-SDS-FAQ(LDLK)_3_	94	5	1	60	40	18	20	200

The fibers were electrospun using Electroris (FNM Ltd., Fanavaran Nano-Meghyas Company, Tehran, Iran,), an electrospinning device with humidity and temperature controllers. The solutions were loaded in a syringe (diameter *d* = 8.7 mm, Terumo) and placed in the horizontal direction. A Gamma high-voltage research power supply (ES50P-10W, Gamma High Voltage Research, Inc., Ormond Beach, FL, USA) was used to charge the solution in the syringe with a positive DC voltage. The positive electrode was connected to the 22G needle (diameter *d* = 0.7 mm) of the syringe and the ground electrode was attached to the collector. A controllable syringe pump (Harvard Apparatus Model 44 Programmable Syringe Pump) in the range of 0.01–100 ml h^−1^ was used to feed the needle. Random and aligned fibers were collected on a flat target and rotating drum covered by aluminum, respectively. The applied parameters were voltage tension = 17/18 kV, tip-collector distance = 20 cm, flow rate = 200/400 μl h^−1^, humidity = 30%, temperature = 22 °C, and rotating rate = 2000 rpm (for aligned fibers).

### 2.2 Characterization

#### 2.2.1 Scanning electron microscopy (SEM)

SEM imaging was conducted with a Tescan VEGA TS 5136XM (TESCAN Company, Brno, Czech Republic) to investigate the samples' morphology. All samples were sputter-coated with a nominally 10 nm thin gold film using a Quorum Tech Q150R S (Quorum Company, East Sussex, UK) sputter coater. The fiber diameters, fibrous and bead contents, and directionality were measured using ImageJ 1.52a.^28,29^

#### 2.2.2 Porosity

Pore size was measured by ImageJ 1.52a. The apparent density and porosity of electrospun fibrous mats were calculated using the following equations, where the thickness of the fibrous mats was measured by SEM:1
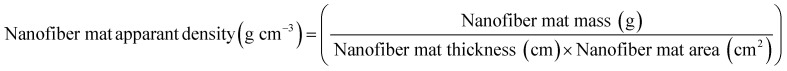
2
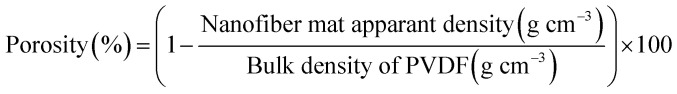


#### 2.2.3 Fourier transform infrared spectroscopy (FTIR)

FTIR spectra were recorded on a PerkinElmer FTIR spectrometer (PerkinElmer, Waltham, MA, USA) in the spectral range 400–4000 cm^−1^ with a resolution of 1 cm^−1^ in transmission mode. The FTIR measurements were repeated three times at random locations for each scaffold type to minimize error. To minimize the interference of the solvents' peaks, entire samples were vacuum-dried overnight. Data processing was performed using Origin 2020 software (OriginLab Corporation, Northampton, MA, USA). All the measured spectra are background-corrected and normalized. A peak analyzer was used to perform non-linear fitting of the peaks in the spectral data. Baseline corrections were performed using a second derivative (zeroes) method to find anchor points and detect the baseline. Hidden peaks were also detected in the spectral range 700–925 cm^−1^ and 925–1350 cm^−1^ by a second derivative method followed by smoothing with the ten-point Savitsky–Golay function with 2nd order polynomial. The deconvoluted spectral peaks were fitted with the Gaussian function. The positions and the number of the components (used as an input file for the curve-fitting function) were obtained from both the second derivative and the deconvoluted spectra. The quality of the fitting was estimated by standard deviation.

Since the peak at 840 cm^−1^ can be assigned to the β, γ, or both phases and 763 cm^−1^ is attributed to the α phase, the relative fraction of the electroactive β and γ phases (*F*_EA_) in terms of crystalline components in any samples can be quantified with the following equation:^30^3
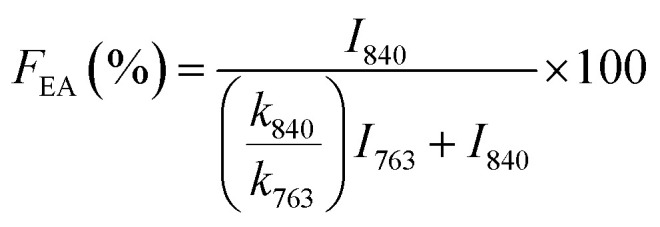
where, *I*_840_ and *I*_763_ are the absorbencies at 840 and 763 cm^−1^ respectively; *K*_840_ and *K*_763_ are the absorption coefficients at the respective wave numbers, whose values are 7.7 × 10^4^ and 6.1 × 10^4^ cm^2^ mol^−1^, respectively. Note that this equation implies the sample is composed only of α and β phases and that the γ phase resonance also overlaps the β phase in this region. The quantification of individual β and γ phases can be performed by using the absorbance of the two bands 1275 and 1234 cm^−1^. However, a much more reliable method is proposed by calculating the peak-to-valley height ratio between the peaks around 1275 and 1234 cm^−1^ and their nearest valley, as illustrated in the equations below^30^4
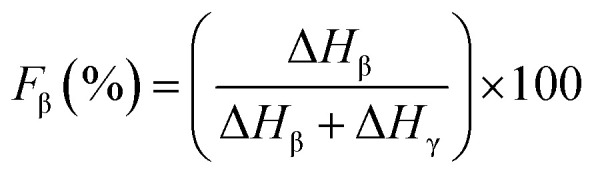
5
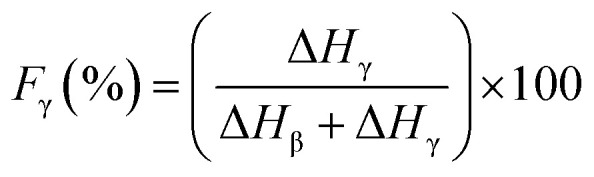
where, Δ*H*_β_ and Δ*H*_γ_ are the height differences between the peak at ∼1275 cm^−1^ and the nearest valley at ∼1260 cm^−1^, and the peak at ∼1234 cm^−1^ and the nearest valley at ∼1225 cm^−1^, respectively. In summary, with FTIR we can determine the relative proportion of α, β and γ phases.^30^

#### 2.2.4 Differential scanning calorimetry (DSC)

DSC data were recorded on a STARe DSC analysis system (Mettler Toledo) equipped with low-temperature apparatus and calibrated with high-purity indium. The experiments were run under a nitrogen atmosphere in standard 40 μL Al pans. DSC measurements were performed between −80 °C and 300 °C at 10 °C min^−1^ on samples having masses of about 5 mg. The degree of crystallinity (*X*_c_) was estimated by using a melting enthalpy (Δ*H*°) of 104.6 J g^−1^ for a 100% crystalline PVDF.^31^

#### 2.2.5 Water contact angle

The contact angle analysis was performed using an in-house contact angle setup, consisting of a camera (Fastcam Nova S6, Photron) with Tokina AT-X PRO D (100 mm F2.8 MACRO) as an optical lens and backlight illumination. Contact angles were measured by dispensing water with a syringe pump (Harvard Apparatus, Pump 11 Pico Plus Elite) at a rate of 10 μl min^−1^, with drop volumes in the range of 5–10 μl. Obtained images and videos were analyzed with ImageJ 1.52a.

#### 2.2.6 Piezoelectric response test

**Fig. 1:**
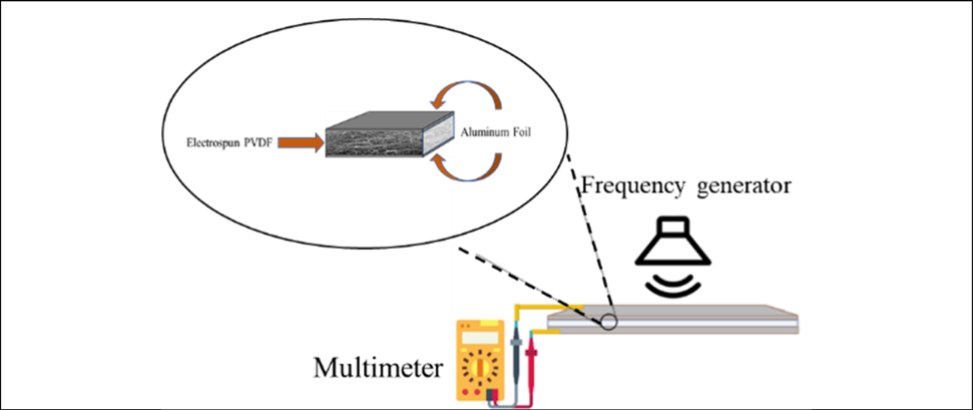
Schematic of piezoelectric response test. Stimulated by different frequencies.

To measure the voltage generated upon sound stimulation, the electrospun membranes with sizes of 2.5 cm × 4 cm were sandwiched between two aluminum foils as an electrode. A copper wire was attached to the electrode on each side to provide a connection with a multimeter (Fluke® 175 True-RMS Digital Multimeter). The membranes were covered with a thin layer of transparent Polyethylene Terephthalate (PET). To demonstrate the piezoelectricity of PVDF-based membranes, the degrees of sample electrical response were qualitatively measured as the samples were stimulated with different sound frequencies from 1 to 2000 Hz. The piezoelectric output was measured as the resulting voltage over frequency. Controls were performed by measuring the voltage with no external force applied. Fig. 1 shows the schematic of the piezoelectric response test.

#### 2.2.7 Culturing murine neural stem cells (mNSCs)

mNSCs were isolated from the striatum of 8-week-old CD1 albino mice, specifically from the Sub-Ventricular Zone (SVZ). The hNSCs were cultured in T75 flasks at a density of 1 × 10^4^ cells per cm^2^, utilizing a serum-free medium supplemented with basic fibroblast growth factor (10 ng per ml bFGF) and epidermal growth factor (20 ng per ml EGF). The cells were maintained in a humidified incubator at 37 °C, 5% O_2_, and 5% CO_2_. Once the neurospheres reached a diameter of 100–150 μm, they were mechanically dissociated by pipetting to transition to a single-cell state.

#### 2.2.8 *In vitro* cellular differentiation

Cells were cultured on diverse electrospun scaffolds, which were pre-attached to the bottom of a 48-well plate (Corning® Costar® TC-Treated Multiple Well Plates). The cell density was set at 3 × 10^4^ cells per cm^2^ in a fresh basal medium supplemented solely with FGF (10 ng ml^−1^). For neuronal and glial differentiation, after 2 days *in vitro* (DIV), the medium was gradually substituted with a medium containing leukemia inhibitory factor (LIF, 20 ng ml^−1^) and brain-derived neurotrophic factor (BDNF, 20 ng ml^−1^) to facilitate the differentiation of cells toward three neuronal and glial populations in hNSC progeny.

As a positive control, CULTREX-BME® (R&D Systems) was utilized. It was positioned in wells devoid of scaffolds and incubated overnight. Following this, the Cultrex was aspirated and substituted with bFGF media.

##### 2.2.8.1 Cell viability

After 7 DIV of differentiation, cell proliferation was assessed *via* CellTiter 96® Aqueous One Solution Cell Proliferation Assay (MTS assay, Promega, Madison, WI, USA): MTS solution was added to the culture media (1 : 5) and incubated for 1 h at 37 °C. The supernatant of each sample was quantified *via* Infinite M200 PRO plate reader (Tecan, Männedorf, Switzerland) by measuring absorbance at 490 nm.

#### 2.2.9 Immunofluorescence

After 7 DIV, cells were fixed using paraformaldehyde (PFA, Sigma-Aldrich 95%) following this protocol: a 5 minute wash in PBS, fixation with 2% PFA for 10 minutes, followed by an additional fixation with 4% PFA for 10 minutes. The PFA solutions were diluted in PBS. After two 5 minute washes in PBS, cell membranes were permeabilized by treating the cells with 0.3% Triton X-100 (Sigma-Aldrich) for 10 minutes at 4 °C. To block nonspecific binding sites, cells were then exposed to 10% normal goat serum (NGS, GIBCO) for 1 h at room temperature. After three washes in PBS, primary antibodies were diluted in a buffer composed of PBS, 1% NGS, and 0.3% triton and applied overnight at 4 °C. Specifically, Glial Fibrillary Acidic Protein (GFAP) was used to label astrocytes, βIII-Tubulin (βIII-TUB) for neurons, and Galactocerebroside (GALC) along with the oligodendrocyte marker (O4) for oligodendrocytes. Following three 5 minutes of washes in PBS each, secondary antibodies were diluted in the same buffer used for the primary antibodies and applied for 2 h at room temperature in the dark. Subsequently, the cells were again washed in PBS three times for 5 minutes each. Hoechst (diluted at 1 : 500 in PBS) was then applied for 10 minutes in the dark to label cell nuclei. After a 5 minute wash in PBS and Milli-Q water, FluorSave™ (Millipore) reagent was applied to preserve immunofluorescence. It's noteworthy that solutions for GALC-O4 were prepared without 0.3% Triton. Both assays were conducted in triplicate. A minimum of three randomly selected fields for each independent experiment were imaged at 20× magnification using a Zeiss Microscope with Apotome System for staining. Cell quantification was performed by manually counting positive cells for each marker using NIH-Fiji software. Additionally, measurements were taken for the length of axons and the soma area of astrocytes. Colored images were transformed into binary images (black and white, 8-bit), and adjustments were made using the automated threshold algorithm with threshold values ranging from 0 to 255. After isolating a single cell, the scale was established, the perimeter traced, and the software automatically computed the defined area. Following this, the length of the axons was measured by setting the scale and tracing the axon. An additional valuable tool employed was the ‘Directionality’ automatic orientation calculator, which provided the frequency distribution of each orientation.

#### 2.2.10 Statistical analysis

Data was processed using Excel, GraphPad Prism 8, and OriginPro 1.52a software. Reported values are as means ± standard error of the mean (SEM). All experiments were repeated three times. Secondary structures were analyzed using One-way ANOVA (paired comparison plot), and Tukey's post hoc test was used for comparative analysis and statistical significance, delineated as *p ≤ 0.05, **p ≤ 0.01, and ***p ≤ 0.001. The MTS assay was processed through one-way ANOVA followed by Dunnett's multiple comparison tests. For *in vitro* studies, the βIII-TUB and GFAP were evaluated by two-way ANOVA followed by Bonferroni's multiple comparison test, and GalC-O4 was performed *via* one-way ANOVA followed by Dunnett post-test. The axon's length and soma's area were evaluated by ordinary one-way ANOVA followed by Tukey's multiple comparison test.

## 3. Results and discussion

Initially, the physical characteristics of PVDF scaffolds were investigated through a suitable tissue engineering substrate lens. With insight from previous studies, the correlation of fiber morphology (fibrous and bead content), orientation, and additives on the electroactive phase content of electrospun PVDF mesh were examined, along with cell survival and differentiation.^30^ They demonstrated that a lower bead content and the most uniform medium-range fiber thickness, consequently resulting in the highest content of the electroactive phase and fiber alignment, have a profound effect on neuronal cell alignment.^30^

### 3.1 Characterization of fiber morphology, porosity, directionality, and contact angle

**Fig. 2:**
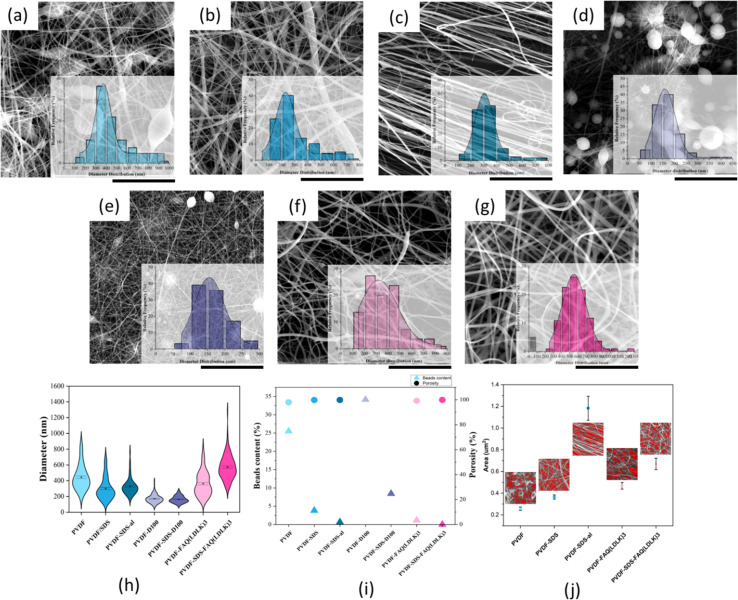
SEM micrographs along with diameter distribution of electrospun PVDF scaffolds with and without additives: (a) electrospun PVDF scaffold fabricated by DMF/acetone (60 : 40) solution shows non-uniform fibers including spindle shape beads and fibers with the average diameter of 444.4 ± 15.6 nm, (b) PVDF-SDS, electrospun scaffolds with more uniformity of fibers after adding a surfactant and average diameter of 300.6 ± 13.1 nm, (c) electrospun PVDF-SDS-al scaffold comprises defect-free fibers with highly-alignment shows thinner fibers about 328.1 ± 11.1 nm (d) electrospun PVDF made of 100% DMF solution reveals round shape beads and very thin fibers, (e) electrospun PVDF-SDS scaffold made of 100% DMF solution contains fewer beads and more fibrous content (f) PVDF-FAQ(LDLK)_3_ electrospun scaffold including SAPs reveals defect-free and uniform fibers with average diameter of 361.7 ± 14.7 nm, (g) PVDF-SDS-FAQ(LDLK)_3_ electrospun scaffold contains SDS and SAPs with very uniform fibers and lowest bead content but higher average diameter 572.3 ± 14.8 nm, (h) Violin plot of the diameter distribution of electrospun scaffolds, (i) porosity and bead content plot, (j) pore size. Scale bars present 20 μm.

Random and aligned scaffolds were fabricated by electrospinning 25% (w/v) PVDF in DMF:acetone (60 : 40 and 100 : 0) solutions with and without SDS and FAQ(LDLK)_3_. All scaffolds excluding those lacking additive and co-solvents exhibited uniform and defect-free fibers with a mean diameter of 200–600 nm, a necessity for the formation and improvement of electroactive phase content as Ico *et al.* showed the negative logarithmic relationship between fiber diameter and piezoelectric constant.^32^ On the other hand, a significant reduction in bead content (including greater α phase) for scaffolds comprising SDS and FAQ(LDLK)_3_ leads to higher electroactivity. This was shown in our previous study that α and β phases are directly related to specific fiber morphologies and that they make up the dominant fraction of bead and fibrous parts of the fibers.^30^ Regarding the scaffolds modified by SAPs, it was revealed that the addition of SAPs can significantly reduce both the bead content and the average fiber diameter (Fig. 2). Therefore, these fiber diameters and morphological patterns may prove ideal both from a purely physical perspective and in the context of piezoelectric output. The increase in fiber diameter for the PVDF-SDS-FAQ(LDLK)_3_ scaffold is due to the formation of a very fine spiderweb-like mesh (<50 nm diameter) due to the existence of SDS and SAP, which is excluded from the average diameter calculation of common electrospun nanofibers.

**Fig. 3:**
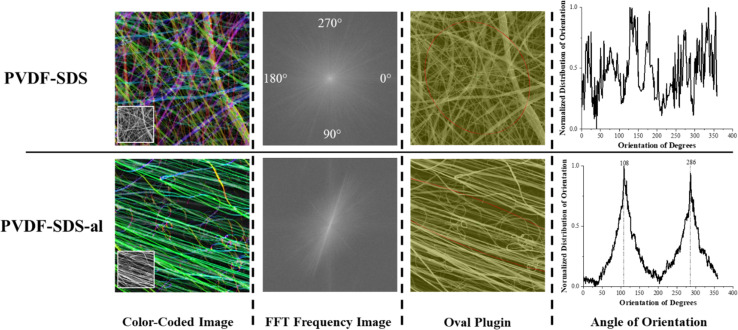
The orientation of fibers was measured on electrospun scaffolds onto a stationary collector and rotating drum (2000 rpm rotating speed). Fast Fourier Transform (FFT) spectra showed a broad distribution of intensities for a stationary collector and two clear peaks for a high-speed rotating collector which are characteristics of highly organized structures. Fiber alignment was significantly increased by using a high-speed rotating collector.

For the aligned fiber scaffold, resultant fibers showed uniform morphology, clear alignment, and a slight decrease in the diameter of the fibers along with an overall narrower diameter distribution. The degree of directionality of the fibers was analyzed as an important function for their potential influence over cell alignment once seed on the scaffold. To evaluate the fiber alignment, derivatives of Fast Fourier Transformations (FFT), color-coded images, and an oval plugin for SEM images were employed. The obtained intensity spectrum of the aligned scaffold showed the peaks related to the main fiber directions at the angles of 108 and 286° (Fig. 3). On the other hand, fiber alignment was quantified as a coherency value by OrientationJ.^28^ The coherency values of aligned and random fibers are 0.673 and 0.084, respectively. Since a higher value indicates a stronger coherent orientation of fibers, the majority of fibers are indicated to be aligned in the PVDF-SDS-al scaffold while PVDF-SDS showed disordered fiber behavior.

All scaffold conditions exhibited estimated porosity values greater than 90%. However, the aligned scaffold showed a slight decrease in estimated porosity. These results were explained by a fiber “packing effect” as the fibers aligned, increasing with the anisotropy degree and confirmed from pore size distributions since average pore size increases as the alignment increases.^33^Fig. 2(j) shows that pore size differed by double for aligned and random fiber mesh (PVDF-SDS), with mean pore size of 1.18 ± 0.11 μm^2^ and 0.36 ± 0.19 μm^2^, respectively. Considering these attributes, the aligned fiber scaffold should promote a higher axon orientation due to its well-aligned fibers. However, its larger pore size may impede cell adhesion and proliferation, providing less fiber area for neurites and cells to extend and spread.^20^ Nonetheless, given the considerably “directional” organization of spinal cord tissue, which includes dendrites, axons, and spinal nerves, aligned fibrous scaffolds have the potential to effectively guide cells and neurites. Despite the considerable variation in the mean fiber diameter typically achieved (ranging from <100 nm to 5 μm), fibrous scaffolds can play a crucial role in this guidance. Notably, there is currently no study that investigates the impact of scaffolds with varying fiber diameters on neurite outgrowth when implanted in animal models of SCI.^34^

Various microscopic characteristics, such as fiber diameter and topography, along with macroscopic features like fiber orientation, can be manipulated to significantly alter the physical properties of fibrous scaffolds. This includes adjusting scaffold porosity, morphology, and architecture. Conversely, the chemical characteristics depend on the inherent properties of the materials used and any chemical modifications applied.

A water contact angle test was conducted to assess the wettability of the scaffold surface. This analysis is crucial because a hydrophilic substrate should better mimic the cellular environment, playing a pivotal role in cell attachment, proliferation, and differentiation.^35,36^ Previous studies have affirmed that alterations in composition can influence the hydrophilicity of 3D-printed scaffolds.^37^ Indeed, it has been shown that cell adhesion can be controlled not only by alterations in nanofibers diameter and porosity but also surface modification of nanofibers.^38,39^

**Fig. 4:**
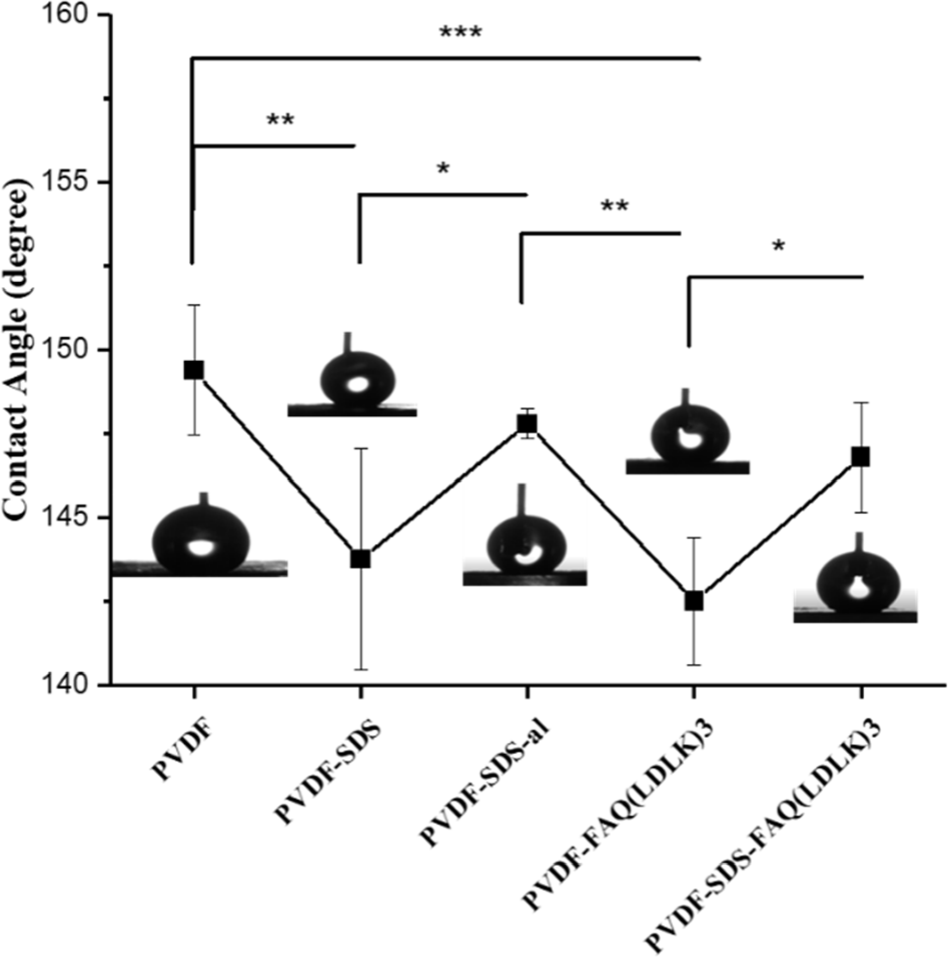
The water contact angle for electrospun scaffolds with different statuses. The line graph shows the reduction of contact angle after adding SDS or SAPs from about 150° to below 145°.

Other research groups reported that increased wettability can accelerate PVDF degradation and anti-inflammatory response.^25,40^Fig. 4 indicates the effect of SDS, SAP modification, and fiber alignment on scaffolds' wettability. It is observed that scaffolds modified by SAPs (142.50° ± 1.91°) and SDS (143.75° ± 3.3°) are more hydrophilic than either PVDF (149.40° ± 1.94°) or PVDF-al (147.80° ± 0.44°) scaffolds. Furthermore, aligned scaffolds show higher hydrophilicity rather than random due to its larger pores.

### 3.2 Electroactive crystalline phases

In its solid state, PVDF is a semi-crystalline polymer which, according to its conformation, can have five different polar and nonpolar polymorph phases (denoted as: α, β, γ, δ, and ε).^41^ The non-polar polymorph α-phase, with anti-parallel stacking of the dipoles, has the lowest energy and is the most stable and favorable phase. However, the β-phase and the γ-phase are the most desirable polymorphs as they exhibit strong electrical dipole moments that are responsible for the enhanced piezoelectric activities.^42^ Gee and colleagues applied the electrospinning method to produce piezoelectric PVDF nanofiber membranes, and their statistical analysis revealed that the solvent ratio and flow rate are the primary factors affecting the β-phase fraction.^43^ Additionally, research by Khalifa and colleagues explored the combined impact of electrospinning and nanofillers, finding that these elements significantly enhance the β-phase fraction.^44^ During electrospinning, the stretching of the electrospun jet in samples containing SDS and SAPs facilitated the formation of the β-phase, while decreasing the α-phase and increasing the β- and γ-phase contents. As a result, the β- and γ-phase are particularly notable for their superior piezoelectric properties.^45^

**Fig. 5:**
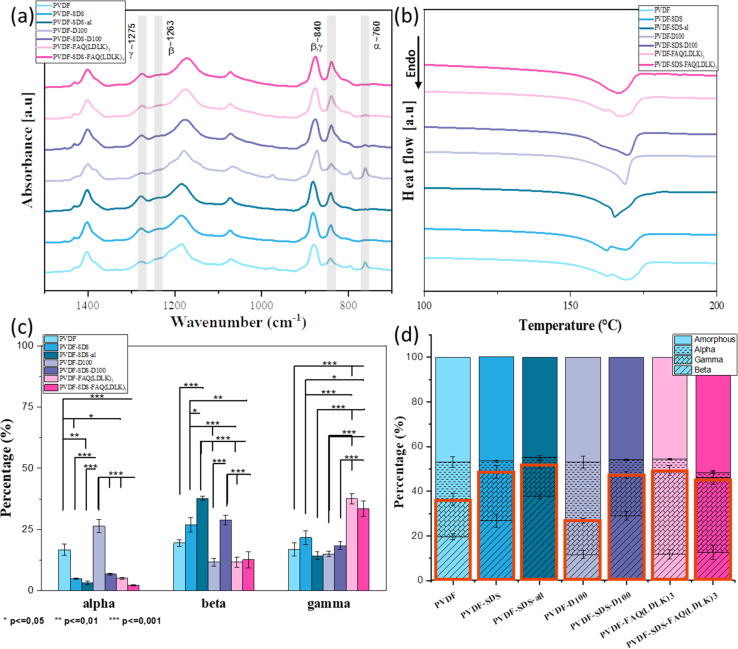
(a) FTIR spectra along with the specific absorption peaks corresponding to the α, β, and γ to measure phase content, (b) DSC thermogram showing melting peaks to measure the degree of crystallinity of scaffolds, (c) phase content measured by the three mathematical resolution enhancement methods of Fourier self-deconvolution, second derivative analysis, and band curve-fitting of FTIR spectra. (d) Bar charts representing percentages of each phase and the electroactive phase are determined by merging phase content (FTIR) and degree of crystallinity (DSC) results. The orange box indicates the overall electroactive phase content. Data are represented as average ± SEM (*N* = 3). Statistical analysis: one-way ANOVA followed by Tukey multiple comparison tests. Statistical analysis shows significant differences between conditions (*p ≤ 0.05, **p ≤ 0.01, and ***p ≤ 0.001).

Since IR spectroscopy can discriminate between the various crystalline phases of the PVDF, it was used to establish the proportion of polymorph phases of PVDF scaffolds. The vibrational peaks observed at 1071, 1176, and 1397 cm^−1^ are assigned to bending vibration C–C, swinging vibration CH_2_, and motion vibration CF_2_ group of PVDF respectively. The specific spectral regions corresponding to the α, β, and γ are between 900 - 700 cm^−1^ and 1250–1200 cm^−1^. The vibrational peaks observed at 760 cm^−1^ are attributed to the non-polar crystalline α-phase. The nucleation of the fully polar β-phase in the PVDF scaffolds is shown from the appearance of the intense vibrational band at 1263 cm^−1^. The semi-polar γ-phase is observed at the 1275 cm^−1^ peak. The characteristic peak at 840 cm^−1^ also indicates β and γ-phase formation in the PVDF scaffolds. Fig. 5(a) shows the IR spectra of all samples. Deconvolution and second-derivative procedures were used to facilitate finding the peak positions and intensity of α, β, and γ in IR spectra and quantitative analysis of the phase content. The quantitative results on phase content were measured by curve-fitting and peak deconvolution (ESI Fig. 1 and 2). Fig. 5(c) and (d) displays the percentages of phase content. By comparing the phase content, we observed a decrease in α phase and an increase in the β- and γ-phases content when SDS and SAPs were added into the electrospinning solutions, as indicated in Fig. 5(c). Although modified scaffolds show improvement in the electroactive phases, the fiber-aligned scaffold demonstrated significant phase modification: the aligned scaffold shows a significant increase in the β phase (37.7%) while the α phase reduced from 4.9 to 3.2%. It can be due to the extra stretching force applied by high-speed rotation collectors that resulting in further molecular chain conformation and orientation. The high-speed collector influenced directly the increase of electroactive phases by uniaxially stretched molecular chains, resulting in a higher degree of molecular orientation which promotes the formation of the dipoles.^33^ The increase of β-phase in additive-modified scaffolds is attributed to the ionic bond between positive and negative charges of additives and fluorine (−) and hydrogen (+) atoms of PVDF, which would result in the alignment of dipoles.^46^ In order to determine the relative amorphous-to-crystalline composition of the samples, we studied their melting behavior using DSC. Fig. 5(b) shows first heating DSC thermograms, giving the melting point (*T*_m_) (endotherm peak), enthalpy of fusion (Δ*H*_m_), and degree of crystallinity (*X*_c_) of the samples. These values are summarized in Table 2. Thermogram curves of samples demonstrate broad and double endothermal peaks between 160 and 170 °C that are related to the presence of α and β phases, which confirms the FTIR results. According to previous research, DSC is not used to distinguish these two phases, but to calculate the degree of crystallinity of the samples.^47^ Given the measured degree of crystallinity, the addition of additives and aligned fibers doesn't increase the degree of crystallinity. All information from FTIR and DSC is summarized in the bar chart (Fig. 5(d)). It reveals that, by adding SDS and SAPs or fabricating an aligned scaffold, the electroactive phase increased significantly while the proportion of amorphous and crystal phases remained relatively constant.

**Table 2:** Melting temperature (*T*_m_), melting enthalpy (Δ*H*_m_), and degree of crystallinity (*X*_c_) of electrospun PVDF scaffolds

Samples	Δ*H*_m_ (J g^−1^)	*T*_m_ (°C)		*X*_c_ (%)
PVDF	55.63	162.6	169	53.23
PVDF-SDS	55.55	162.4	168.8	53.16
PVDF-SDS-al	57.65	165		55.17
PVDF-D100	55.52	168.5		53.13
PVDF-SDS-D100	56.51	169.3		54.08
PVDF-FAQ(LDLK)_3_	56.82	162.15	168	54.37
PVDF-SDS-FAQ(LDLK)_3_	50.55	166.77		48.37

### 3.3 Piezoelectric response test

**Fig. 6:**
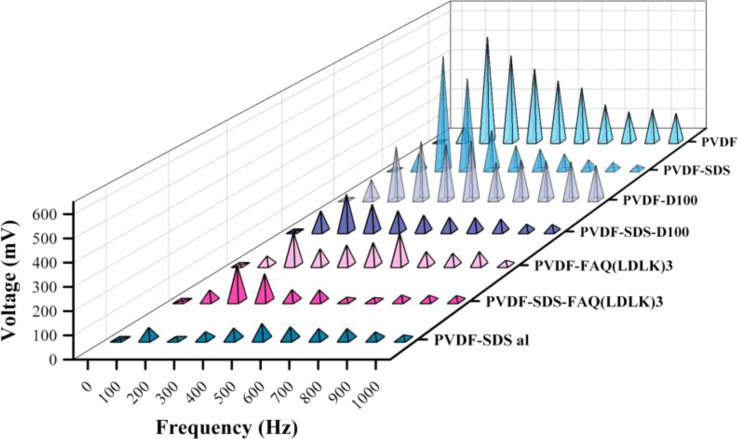
Piezoelectric response test. Stimulated PVDF scaffolds under different frequencies. All samples were subjected to electrical polarization, resulting in the subsequent generation of voltage through frequency vibration stimulation.

As shown in Fig. 6, through the sound-mechanical strain test all the samples have been determined to have piezoelectric response. These results demonstrate that all electrospun PVDF fibrous scaffolds are getting electrically poled by mechanical vibration due to piezoelectric properties. All samples have shown a maximum voltage when stimulated between 100 and 500 Hz frequency. The voltage generated by piezoelectric materials is directly proportional to both the rate of strain (how quickly the material deforms) and the d33 component of the material's dielectric tensor. When subjected to low-frequency sound waves, the film experiences compression and extension at a rate that escalates with increasing frequency. Greater deformation, or the speed at which the film compresses and recovers in response to external forces, coupled with heightened electroactivity, yields a higher output voltage. However, as the strain rate escalates, the film becomes unable to adequately respond to deformation, resulting in a diminishing piezoelectric voltage that eventually reaches zero at higher frequencies. The variation in the maximum charge among the samples can be attributed to the correlation between the thickness of the PVDF mesh and its deformability and recovery under mechanical strain, and the generated voltage. The findings reveal a linear association between mesh thickness and voltage (ESI Table 1). Given the diverse thicknesses and volumetric densities of the meshes, we approached this sound test experiment as a qualitative test of piezoelectricity.

### 3.4 *In vitro* assays

**Fig. 7:**
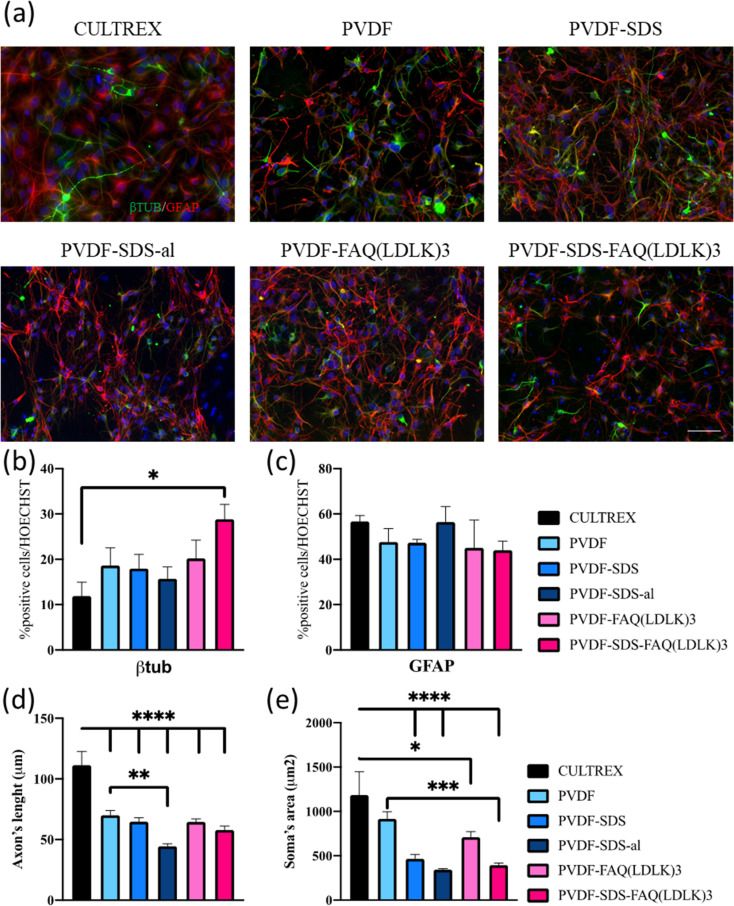
(a) Fluorescence microscopic images were captured of NSCs on PVDF mats, as well as on Cultrex (control), at the conclusion of the 7 days differentiation period. β-Tubulin (βIII-TUB) was stained in green, glial fibrillary acidic protein (GFAP) in red, and cell nuclei in blue. (b) Percentages of βIII-TUB cells. (c) Percentages of GFAP-positive cells. d) Axon's length is expressed in μm. (e) Soma's area is expressed in μm^2^. Scale bar presents 50 μm. Statistical analysis: one-way ANOVA followed by Tukey multiple comparison test. Statistical analysis shows significant differences between conditions (*p ≤ 0.05, **p ≤ 0.01, and ***p ≤ 0.001).

*In vitro* investigations to test cell differentiation and cell behavior were carried on the electrospun PVDF-based nanofibrous mats.^48^ The morphology of differentiated murine neural stem cells (mNSCs) was studied on five different types of electrospun nanofibrous mats (PVDF, PVDF-SDS, PVDF-SDS-al, PVDF-FAQ(LDLK)_3_ and PVDF-SDS-FAQ(LDLK)_3_). The differentiation of NSCs progeny at 7 DIV was assessed by staining neurons with the βIII-TUB marker, astrocytes with GFAP, and oligodendrocytes with GALC/O4. The hNSCs were distributed uniformly after 7 DIV in culture on electrospun PVDF scaffolds and showed a typical polygonal morphology with long cytoplasmic extensions and clearly visible cell–cell contacts. Diverse cell morphologies were observed upon cultivation on PVDF fiber surfaces (Fig. 7). Cells cultured on Cultrex displayed a robust bidirectional alignment, predominantly adopting a bipolar morphology (Fig. 7). In contrast, NSCs cultured on PVDF showcased a polygonal structure, demonstrating a pronounced inclination to align along the material fibers (Fig. 7), as highlighted by elongated cytoplasmic extensions. Glial cells exhibited a stretched and radiant network of dendrites, while neuronal cells displayed elongation along a distinct axis.

**Fig. 8:**
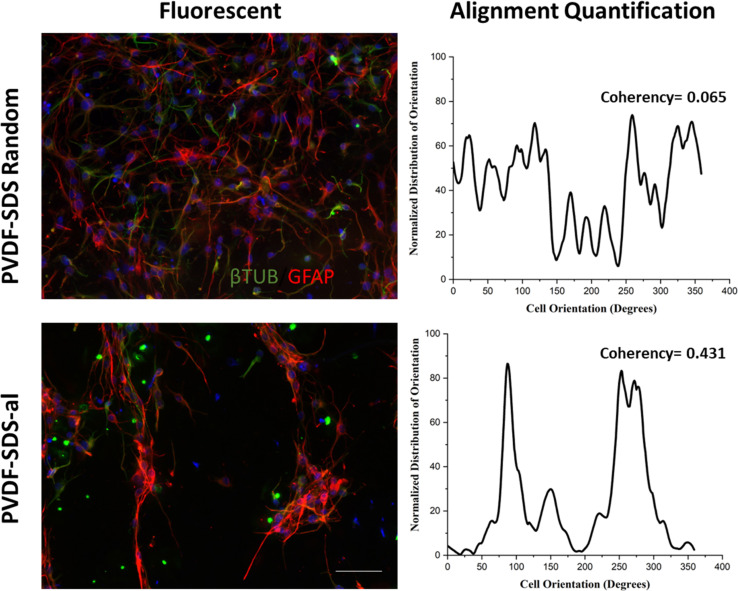
Nanofibers alignment regulates neuronal cell orientation. Fluorescence images show a disordered spread of cells on the random scaffolds (PVDF-SDS). In contrast, cell growth on the aligned nanofibers (PVDF-SDS-al) shows an ordered pattern. Entire visual outcomes were proved by alignment quantification, two peaks at 85 and 265° for PVDF-SDS-al scaffold, and coherency values (much higher coherency value for PVDF-SDS-al rather than PVDF-SDS random scaffold. Scale bar presents 50 μm.

Based on the data presented in Fig. 7, βIII-TUB served as a marker indicating the differentiation into neurons of NSCs. Upon comparison with Cultrex, NSCs efficiently differentiate into neuronal cells when cultured on PVDF-SDS-FAQ(LDLK)_3_. This could be due to the modification of the surface chemistry of the scaffold by the addition of both SDS and SAPs, which resulted in increased hydrophilicity, functionalization with the FAQ pro-neuronal motif^49^ and subsequently cell attachment. In the context of the GFAP marker, all the samples exhibited comparable results to Cultrex, with no significant differences observed in the immunostaining of GFAP. As for the length of axons, a statistically significant difference was observed between electrospun PVDF scaffolds and the control, Cultrex. Additionally, GFAP staining was employed for the analysis of the soma's area. In electrospun PVDF scaffolds treated with SDS, the soma's area of GFAP-positive cells exhibited a significant difference compared to Cultrex. It is crucial to emphasize that the size, distribution, and interconnectivity of pores play a pivotal role in governing the diffusion rates of nutrients and the removal of waste. A substrate with an optimal pore size not only facilitates cell seeding and penetration but also enhances oxygen diffusion and distribution within the scaffolds. Furthermore, pore size significantly influences cell adhesion, intercellular interactions, and cell spreading. Neurons have the ability to sense the scaffold surface, and focal adhesions play a regulatory role in signaling complexes and integrin function. This triggers a signaling cascade that promotes cell proliferation and differentiation.^50,51^ Moreover, to corroborate these data, cell viability studies were performed using MTS assay. Cell proliferation and viability were assessed with an MTS assay (ESI Fig. 3). Cells seeded on gold standard Cultrex produced comparable values when compared to treated samples. In the case of the aligned scaffold, we note that the reduction in axon length and soma area size was predictable due to the increasing pore size and the corresponding reduction in the area available for cell spreading. The effectiveness of PVDF meshes as a potential therapy needs to be assessed, especially concerning neural connectivity. Understanding how these scaffolds impact neural connectivity is essential for evaluating their potential to promote cell proliferation and differentiation in the context of neural therapy. On the other hand, as shown in Fig. 8, neuronal cells exhibited irregular morphology on the prepared random PVDF-SDS. In comparison, the growth of neuronal cells on the aligned scaffold (PVDF-SDS-al) is orientated at 85°. Cell orientation was quantified as a coherency value of aligned and random scaffolds that are 0.431 and 0.065, respectively, which proved a stronger coherent orientation of fibers for cell growth on aligned scaffolds. These results imply that using an aligned scaffold guides cell growth.

Ensuring continuity in the transmission of information is crucial for regaining lost locomotor performance. Therefore, the quantitative evaluation of the evolution of neural processes' directionality over time was essential.^52^ The materials and methods provided a comprehensive description, illustrating a side-by-side comparison of the mean directionality in Cultrex (used as control) and electrospun PVDF meshes.

**Fig. 9:**
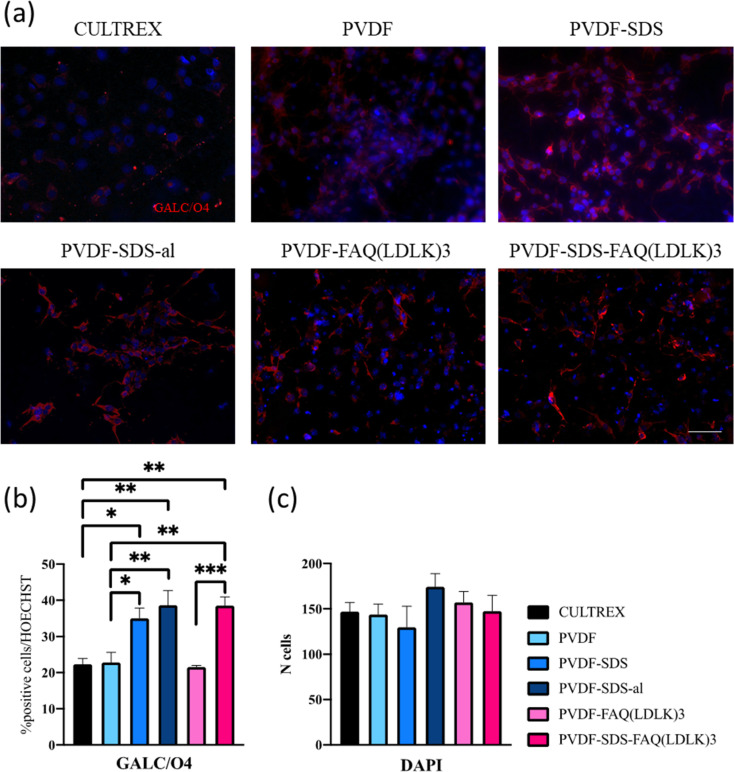
(a) Fluorescence microscopic images of hNSCs on PVDF mats, as well as Cultrex (control) at the end of the 7 days differentiation period. GALC/O4 were stained in red and nuclei in blue. (b) Percentages of GALC/O4 positive cells. (c) Numbers of cell nuclei counterstained with DAPI. Scale bar presents 50 μm. Statistical analysis: one-way ANOVA followed by Tukey multiple comparison test. Statistical analysis shows significant differences between conditions (*p ≤ 0.05, **p ≤ 0.01, and ***p ≤ 0.001).

Ultimately, the percentage of oligodendrocytes was assessed, as indicated in Fig. 9, employing GalC-O4 markers. In this instance, notable differences were observed among Cultrex and electrospun scaffolds in terms of oligodendroglial differentiation suggesting a significant impact of scaffold composition on NSCs differentiation.

Specifically, Cultrex displayed the lowest oligodendrocyte percentage compared to PVDF-SDS, PVDF-SDS-al, and PVDF-SDS-FAQ(LDLK)_3_ highlighting the importance of scaffold material in guiding cellular differentiation. One relevant paper by Thorrez *et al.*,^53^ discusses the differentiation of NSCs into oligodendrocytes on poly(l-lactide) (PLLA) nanofiber scaffolds. While the scaffold material differs from the ones mentioned in the excerpt, the underlying principles of scaffold influence on oligodendroglial differentiation are likely to be relevant. Another noteworthy distinction in oligodendrocyte percentage was observed between PVDF and PVDF-SDS. It is worth noting that in samples with the highest oligodendrocyte percentage, SDS was present. This suggests that the addition of SDS (and overall hydrophilicity of the scaffold) may contribute to the production of fibers more conducive to the oligodendroglial differentiation of NSCs. This observation aligns with previous research indicating the importance of scaffold properties, such as hydrophilicity, in directing cellular behavior and differentiation.

Various tissues necessitate specific microenvironments to support fundamental processes such as cell–cell interaction, migration, proliferation, differentiation, and regeneration. In particular, materials derived from PVDF have proven effective as substrates for neuron attachment, growth, and differentiation, with their properties influenced by electrical activity.^33^

In the field of neural tissue engineering, electrospun fibrous mats with aligned fibers offer a significant advantage over scaffolds featuring randomly oriented fibers. This advantage is attributed to the spatial guidance provided by highly aligned fibers, which facilitates neurite outgrowth and axonal elongation along specific directions in the attempt to mimic the native nervous tissue, like in the spinal cord.

NSCs, already proved to be crucial for nerve function and repair *in vivo*,^54^ were chosen to evaluate the potential of electrospun PVDF scaffolds *in vitro* as potential neural cell carriers in future therapies for nervous regeneration. The impact of scaffold morphology, including factors such as fiber size, alignment, and porosity as well as structural properties like crystallinity and wettability, is widely acknowledged for its influence on cell morphology, growth, and differentiation.^55^ Lins *et al.* conducted an analysis on the influence of PVDF fiber alignment on both undifferentiated monkey NSCs and the subsequent differentiation into neuronal and glial cells.^33^ The study revealed that while the growth patterns of undifferentiated stem cells and glial cells remained unaffected by fiber alignment, differentiated neuronal cells exhibited an elongated morphology specifically on aligned fibers.

In comparison to previous studies like Lins *et al.*, our work likely contributes to the scientific community by introducing novel elements such as the use of specific additives like SAPs and SDS in the fabrication of PVDF scaffolds. These additives may have been incorporated to modify scaffold properties such as surface wettability and surface chemistry, which could in turn influence cell–scaffold interactions and ultimately cellular behavior. Therefore, our work extends beyond simply assessing the impact of scaffold morphology on NSCs to exploring the effects of scaffold composition and surface properties on NSC behavior, which adds valuable insights to the field of neural tissue engineering and regeneration.

## 4. Conclusion

This study successfully identified and characterized electrospun PVDF scaffolds conducive to nervous cell growth, highlighting their potential for spinal cord injury regeneration therapies. We explored the effects of incorporating SDS and SAPs additives, as well as utilizing different electrospinning techniques (static target and rotating drum collector), on fiber morphology and electroactive phase content.

Our findings revealed that the addition of SDS and SAPs, as an anionic surfactant and bioactive agent respectively, combined with the use of a rotating collector, resulted in uniform, finer fibers with higher alignment and increased hydrophilicity (notably in PVDF-SDS and PVDF-FAQ(LDLK)3 samples). DSC and IR spectroscopy data indicated a significant increase in electroactive phase content with the inclusion of these additives. Furthermore, the rotating collector was particularly effective in inducing β-phase generation in modified PVDF scaffolds. In terms of biological performance, hNSCs seeded on these electrospun nanofibrous scaffolds exhibited satisfactory proliferation, viability, and differentiation compared to a gold standard. The scaffolds supported cell differentiation into the three main neural phenotypes and promoted notable cell sprouting. The tested SAP demonstrated beneficial effects, suggesting that other functionalized peptide molecules could further enhance scaffold multifunctionality, thereby boosting host nervous regeneration and transplanted cell engraftment. Despite the challenge of larger pores potentially hampering cell survival and axon length, aligned scaffolds were shown to promote a higher degree of cell orientation due to the nano- and microfiber alignment. Ultimately, our results pave the way for the development of electroactive, biomimetic fibrous scaffolds with tailored architectures, offering promising applications in neural tissue engineering.

## Data availability

The data supporting this article have been included as part of the ESI.†

## Author contributions

Conceptualization, M. F., A. R. and F. G.; methodology, M. F., and A. R.; SAPs synthesize, M. G. C.; formal analysis, M. F., A. R., G. F., and A. F.; investigation, M. F., A. R., and G. F.; resources, F. G.; data curation, M. F., A. R. and G. F.; writing—original draft, M. F., and A.R.; writing—review & editing, M. F., A. R., A. F., V. S., L. S., S. B., and F. G.; supervision, F. G.; project administration, F. G.; funding acquisition, F. G. All authors have read and agreed to the published version of the manuscript.

## Conflicts of interest

There are no conflicts to declare.

## Supplementary Material

RA-014-D4RA02309A-s001
