# Supplementary material for: Biomimetic electrospun PVDF/self-assembling peptide piezoelectric scaffolds for neural stem cell transplantation in neural tissue engineering
Source: RSC Adv. 2024 Jul 5;14(30):21277–91. doi: 10.1039/d4ra02309a (PMC11225063; doi:10.1039/d4ra02309a)
Supplement: RA-014-D4RA02309A-s001 [file RA-014-D4RA02309A-s001.pdf]

Electronic Supplementary Material (ESI) for RSC Advances.
This journal is © The Royal Society of Chemistry 2024

Biomimetic Electrospun PVDF/Self-Assembling Peptide Piezoelectric Scaffolds for Neural Stem Cell Transplantation in Neural Tissue Engineering

Mahdi Forouharshad[1], Andrea Raspa[1], Giuseppe Fortino[2], Maria Gessica Ciulla[1], Arman Farazdaghi[3],Vlad Stolojan[4], Luca Stendardo[5], Silvia Bracco[5], Fabrizio Gelain[1,6,*]

[1]Center for Nanomedicine and Tissue Engineering (CNTE), ASST Grande Ospedale Metropolitano Niguarda, 20162 Milan, Italy.
[2]Department of Biotechnology and Bioscience, University of Milano – Bicocca, via R. Cozzi 55, 20125 Milano, Italy.
[3]Chemical and Biomolecular Engineering Department, Whiting School of Engineering, Johns Hopkins University, MD, USA.
[4]Advanced Technology Institute, Electrical and Electronic Engineering, University of Surrey, Guildford GU2 7XH, UK.
[5]Department of Materials Science, University of Milano – Bicocca, via R. Cozzi 55, 20125 Milano, Italy.
[6]Institute for Stem-Cell Biology, Regenerative Medicine and Innovative Therapies, IRCCS Casa Sollievo della Sofferenza, 71013 San Giovanni Rotondo, Italy.

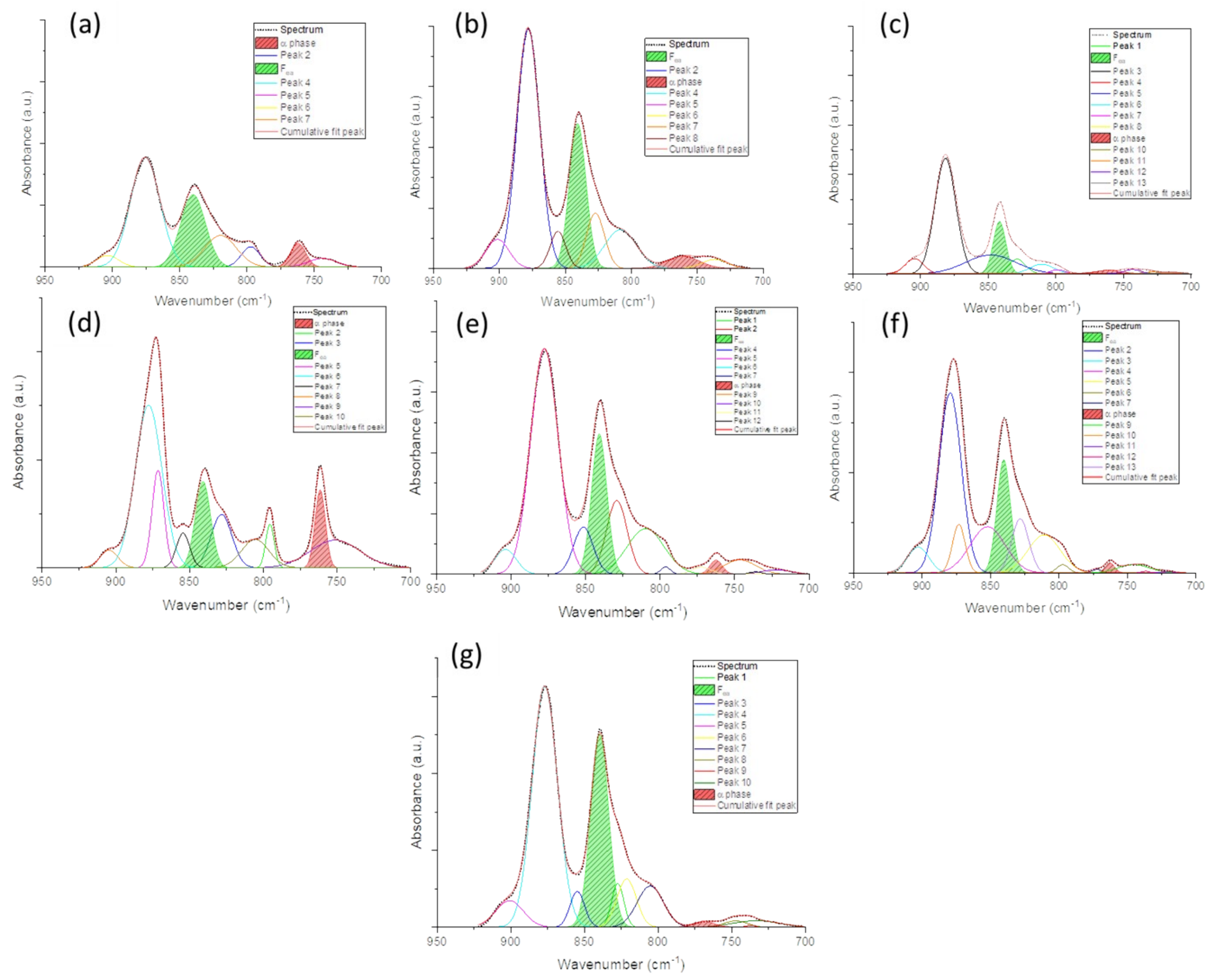

Figure 1. Deconvolution and second-derivative procedures were used to facilitate finding the

peak positions corresponding to the α, β, and γ in their IR spectra (range 950-700 cm^{-1}) and for the quantitative analysis of the phase content. (a) PVDF, (b) PVDF-SDS, (c) PVDF-SDS-al, (d) PVDF-DMF100, (e) PVDF-SDS-DMF100, (f) PVDF-FAQ$(LDLK)_3$, and (g) PVDF-SDS-FAQ$(LDLK)_3$.

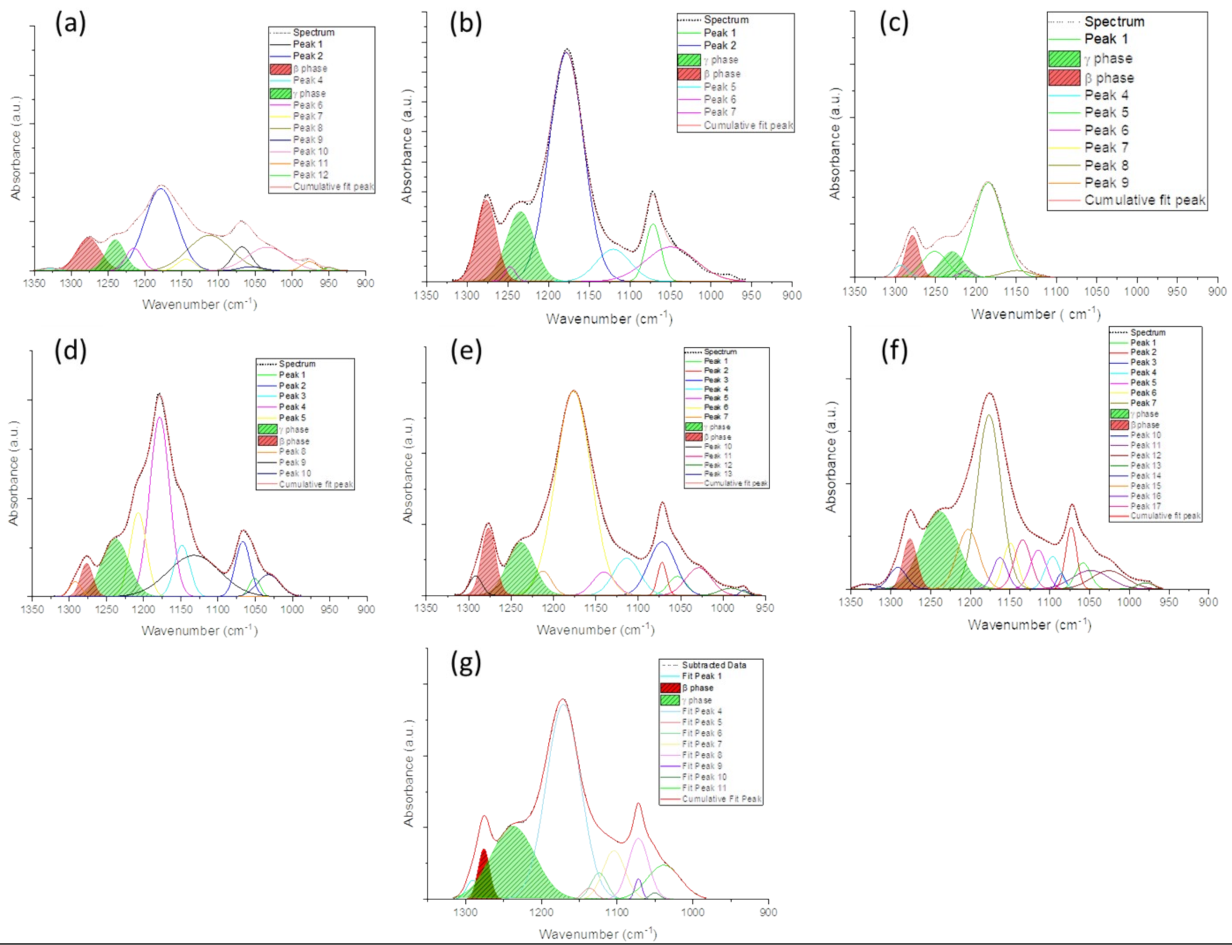

Figure 2. Deconvolution and second-derivative procedures were used to facilitate finding the peak positions corresponding to the β and γ in their IR spectra (range 1350-950 cm^{-1}) and quantitative analysis of the phase content. (a) PVDF, (b) PVDF-SDS, (c) PVDF-SDS-al, (d) PVDF-DMF100, (e) PVDF-SDS-DMF100, (f) PVDF-FAQ$(LDLK)_3$, and (g) PVDF-SDS-FAQ$(LDLK)_3$.

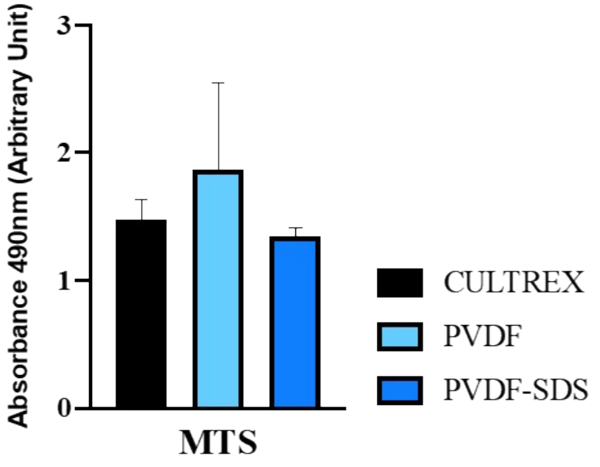

Figure 3. MTS assay on our PVDF and PVDF-SDS samples to assess cytotoxicity. Cultrex, the gold standard, was used as the control. Stem cells were seeded in both the control group and on the PVDF and PVDF-SDS samples. The graph shows no significant differences in cell viability between the PVDF and PVDF-SDS samples and the positive control, Cultrex. As depicted, the cell viability for both PVDF and PVDF-SDS samples closely aligns with the positive control, confirming their biocompatibility.

Table 1. Piezoelectric experiments to investigate the correlation between the thickness of PVDF meshes and the resulting voltage and capacitance.

**Sample (mg/cm²)**	**V_{AC} rms [mV]**	**$V_{p\text{-}p}$ [mV]**	**C_{100Hz} [pF]**	**Θ_{100Hz} [pF]**	**C_{100kHz} [pF]**	**Θ_{100kHz} [pF]**
1	58.22	181.5	375	-89.4	369	-89.4
2	180	532	217	-89.2	210	-89.6
3	625	1780	159	-89.4	154	-89.5
4	38	120	210	-89.3	201	-89.4

V_{AC}: Voltage Alternating Current
$V_{p\text{-}p}$: peak-to-peak voltage
C: Capacitance
Θ: phase shift
